# Use of Next-Generation Sequencing for the Molecular Diagnosis of 1,102 Patients With a Autosomal Optic Neuropathy

**DOI:** 10.3389/fneur.2021.602979

**Published:** 2021-03-25

**Authors:** Majida Charif, Céline Bris, David Goudenège, Valérie Desquiret-Dumas, Estelle Colin, Alban Ziegler, Vincent Procaccio, Pascal Reynier, Dominique Bonneau, Guy Lenaers, Patrizia Amati-Bonneau

**Affiliations:** ^1^University Angers, MitoLab team, UMR CNRS 6015—INSERM U1083, Unité MitoVasc, SFR ICAT, Angers, France; ^2^Genetics and Immuno-Cell Therapy Team, Mohammed First University, Oujda, Morocco; ^3^Departments of Biochemistry and Genetics, University Hospital Angers, Angers, France

**Keywords:** inherited optic neuropathies, mitochondrial disorders, molecular diagnosis, next generation sequencing, retinal ganglia cells

## Abstract

Advances in next-generation sequencing (NGS) facilitate the diagnosis of genetic disorders. To evaluate its use for the molecular diagnosis of inherited optic neuropathy (ION), a blinding disease caused by the degeneration of retinal ganglion cells, we performed genetic analysis using targeted NGS of 22 already known and candidate genes in a cohort of 1,102 affected individuals. The panel design, library preparation, and sequencing reactions were performed using the Ion AmpliSeq technology. Pathogenic variants were detected in 16 genes in 245 patients (22%), including 186 (17%) and 59 (5%) dominant and recessive cases, respectively. Results confirmed that *OPA1* variants are responsible for the majority of dominant IONs, whereas *ACO2* and *WFS1* variants are also frequently involved in both dominant and recessive forms of ION. All pathogenic variants were found in genes encoding proteins involved in the mitochondrial function, highlighting the importance of mitochondria in the survival of retinal ganglion cells.

## Introduction

Inherited optic neuropathies (IONs), the most common cause of underlying blindness of genetic origin, are mitochondrial diseases that cause degeneration of retinal ganglion cells (RGCs) and optic nerves with varying levels of vision loss. Their clinical presentation can be isolated, without extraocular symptoms, or syndromic, in which the optic atrophy is the primary symptom associated with a wide variety of secondary symptoms, mainly of neuromuscular origin (1–3). IONs may be caused either by autosomal dominant variants, leading to dominant optic atrophy (DOA MIM#165500), or by autosomal recessive variants, leading to recessive optic atrophy (ROA), or finally by mitochondrial DNA (mtDNA) variants, causing Leber's hereditary optic neuropathy (LHON, MIM #535000).

The diagnosis of ION is based on three main criteria: (i) reduced visual acuity with a central or cecocentral scotoma; (ii) the presence of a pallor of the optic disk on funduscopic examination, while the retina remains normal; and (iii) the loss of the layer of retinal nerve fibers objectified by optical coherence tomography (4–6). In general, DOA begins in childhood or in young adults, leading to a chronic and slow progression of changes in vision, whereas recessive forms affect younger children more severely (3, 7). Conversely, maternally transmitted LHON leads, in general, to acute loss of vision in one eye, followed by the other eye on average 2 months later.

DOA is mainly caused by pathogenic *OPA1* variants (8–10) and less frequently by mutations in other genes including *OPA3, MFN2, SPG7, AFG3L2, DNM1L*, and *SSBP1* (11–17). These genes are all involved in controlling the mitochondrial dynamics, with the exception of *SSBP1*, which is involved in mtDNA replication. This suggests that the equilibrium between mitochondrial fusion and fission is a key process for the maintenance of RGC physiology. Another gene involved in dominant ION is *WFS1*, the gene responsible for Wolfram syndrome, a recessive condition. *WFS1* is also responsible for DOA associated with neurosensorial deafness (18) and for isolated recessive isolated ION (19). Importantly, *WFS1* encodes a protein of the endoplasmic reticulum that is involved in mitochondrial calcium homeostasis (20).

In addition to *WFS1*, six genes have been identified as responsible for ROA, namely, *TMEM126A, ACO2, RTN4IP1, NDUFS2, MCAT*, and *SLC25A46* (21–26). All these genes encode proteins with a mitochondrial function, but only *SLC25A46* is involved in mitochondrial dynamics (21).

Angers University Hospital (CHU) has provided a national genetic diagnosis for ION for the last 20 years. More than 4,000 DNA samples have now been collected, and an ongoing collection of 800 additional samples is collected each year. Sanger sequencing of *OPA1, OPA3*, and *WFS1*, as well as all LHON mtDNA mutation, was performed for each sample until 2015. Currently, in order to improve the molecular diagnosis of ION, we have designed a resequencing chip targeting 22 ascertained or candidate genes.

Here, we report the results obtained with this ION chip on more than 1,000 DNA samples from individuals affected with ION. This procedure allowed a molecular diagnosis for more than 22% of the cases.

## Materials and Methods

### Patients

The Department of Biochemistry and Genetics at Angers University Hospital (France) is a national center for the genetic diagnosis of ION. DNA samples from 1,102 individuals affected with autosomal ION were collected in this center between 2015 and 2018. All patients had a proven case of clinical optic atrophy, investigated by an ophthalmologist before genetic testing. Where possible, DNA samples were obtained from affected and healthy relatives. Written informed consent was obtained from every individual involved in this diagnosis procedure.

### ION Panel Design, Library Preparation, and Sequencing

The patients were initially screened for the three primary LHON mutations or for all mtDNA genome.

An amplicon library of the target exons was designed with the Ion AmpliSeq Designer (http://ampliseq.com) for the most genes reported to be responsible for ION, or candidate genes identified by whole-exome sequencing. These candidate genes do not have a direct mitochondrial function, but these are selected after analysis of the WES data of other NOH patients by filtering with a list of 1,600 mitochondrial genes. Four primer pools (125- to 275-bp amplicon target sizes) were designed to amplify 313 and 274 amplicons, covering 22 genes (Supplementary Table 1) (12) of a total length of 55.28 and 50.99 kb.

Genomic DNA of patients was extracted from peripheral blood. The Qubit dsDNA High Sensitivity Assay Kit (Thermo Fisher Scientific) was used to quantify DNA for next-generation sequencing (NGS) library construction. Library preparation for each sample was performed using Ion AmpliSeq technologies, and all sequencing data were processed using a dedicated bioinformatics pipeline, which includes three steps: variant calling, annotation, and prioritization (12, 27). The calling module uses a consensus-based approach and combines the prediction of six callers (VariantCaller included with the Torrent Suite, GATK Unified Genotyper, VarScan2, SNVer, LoFreq, and Platypus). All the generated variant calling formats are normalized and decomposed before launching the annotation–prioritization module, which combines NCBI Variant Reporter and ANNOVAR. These tools allow including genomic databases, clinical databases such as CLINVAR, and precomputed results of several prioritization tools (e.g., SIFT, PolyPhen2, LRT, MutationTaster).

### Causative Variant Prioritization

For each patient, we looked for causative variants using the following prioritization strategy: first, we checked for reported pathogenic variants in ION genes, and then we looked for novel loss-of-function (stop–gain, frameshift, and splicing) or novel missense variants in these genes, and eventually, we selected new variants in candidate genes, which should be classified as being of “uncertain significance” according to the American College of Medical Genetics and Genomics, until additional evidence is supporting their pathogenicity.

All dominant variants were absent or had a minor allele frequency (MAF) threshold of <0.0001 in public databases, whereas recessive variants were considered with an MAF threshold <0.005. For familial cases, we specifically selected the variants that matched the inheritance pattern predicted from the pedigrees.

### Sanger Sequencing Validation and Family Segregation Assessment

All putative variants identified by NGS were confirmed by Sanger sequencing, and their segregation was studied in all available members of the family. Primers were designed using the Primer3 software (28). To ensure the quality of Sanger sequencing, amplicons were designed to have a boundary of around 100 bp away from the variant.

## Results

### NGS Quality Results

DNA samples were primarily screened for the presence of one of the three primary LHON mutations: m.3460G>A, m.11778G>A, and m.14484T>C, and excluded from this study if the positive. Ion AmpliSeq NGS technology using autosomal ION panel was performed in a cohort of 1,102 individuals. Only one affected individual was studied per family. Within the design region, 95% of bases had coverage of >25×, >50×, and >100×, indicating that a sufficient coverage was achieved to enable high variant detection sensitivity.

Sanger sequencing was also performed for regions that are not or are poorly covered by the chip sequencing. This concerned specific regions of the following genes: *AFG3L2* (exon1), *SPG7* (exon 1, 13, and 17), *ACO2* (exon 3), *MFN2* (exon 9), *RTN4IP1* (exon 2), *WFS1* (exon 8), and *OPA1* (exon 24).

### NGS Results of the ION Panel

**Figure 1:**
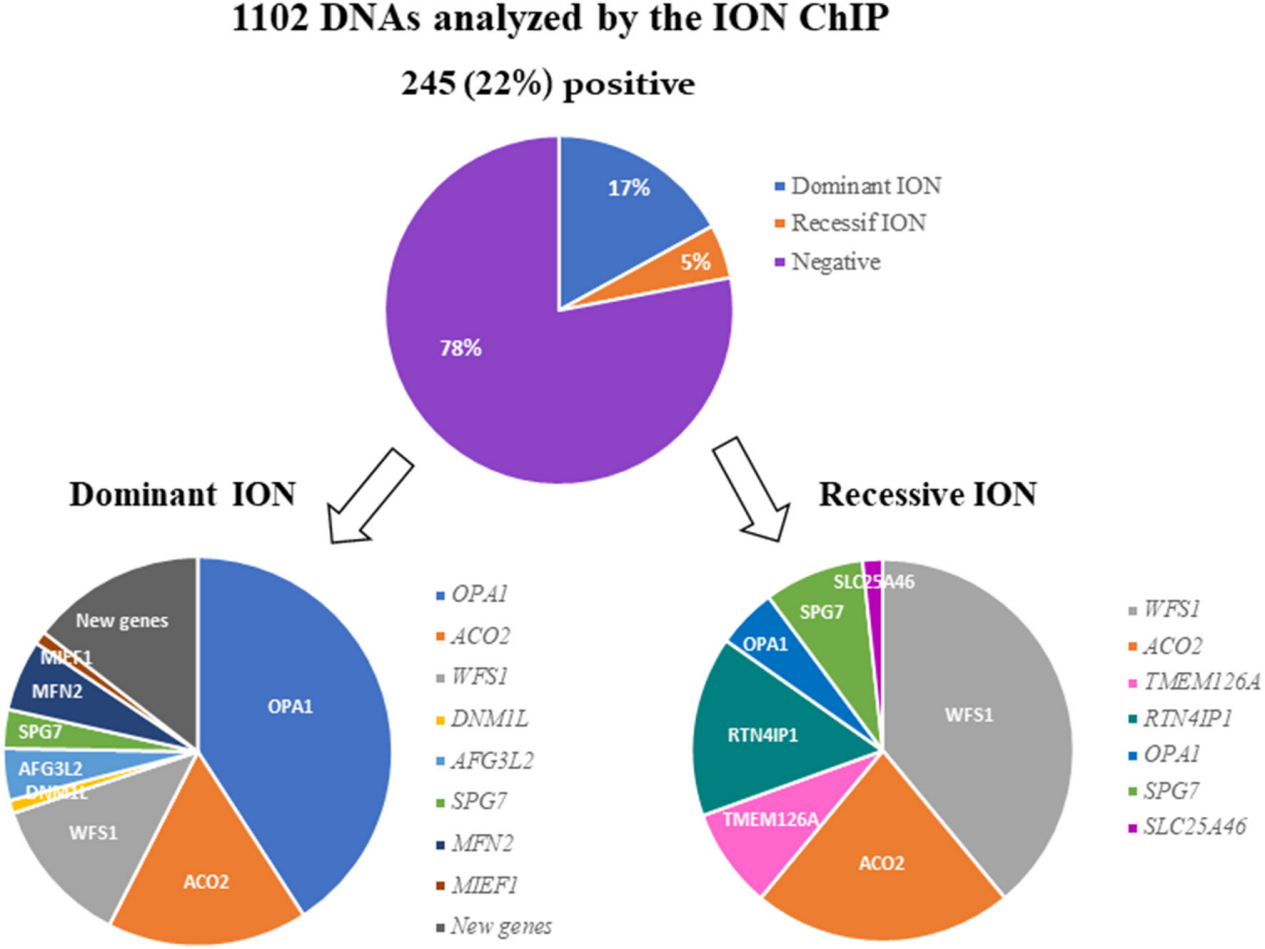
Results of the molecular analysis of 1,102 individuals with ION, using the 22-targeted-gene ION panel. A pathogenic variant was found in 245 (22%) of the DNA samples analyzed. The variants were inherited dominantly or recessively in 186 and 59 cases, respectively. The genes involved in dominant and recessive inheritance are indicated.

After filtering, annotation steps, and variant prioritization procedures, we identified pathogenic variants in 245 individuals (22%) (Supplementary Table 2), including 186 cases (17%) with a dominant mode of inheritance and 59 cases (5%) with a recessive mode of inheritance (Figure 1).

### Dominant Genes

Out of the 186 individuals affected with DOA, 76 (40.86%) harbored a pathogenic variant in *OPA1*. The two other genes most involved in DOA were *ACO2* and *WFS1*, with frequencies of 16.67% (31 cases) and 12.37% (23 cases), respectively.

Among the other genes, we identified variants in *AFG3L2* in 8 individuals (4.3%), in *SPG7* in 6 individuals (3.23%), in *MFN2* in 11 individuals (5.91%), and in *DNM1L* and *MIEF1* in 2 individuals each (1.08%). Lastly, we identified five new candidate genes in 27 families (data not shown), for which dominant variants were found to segregate with the disease (Figure 1).

**Figure 2:**
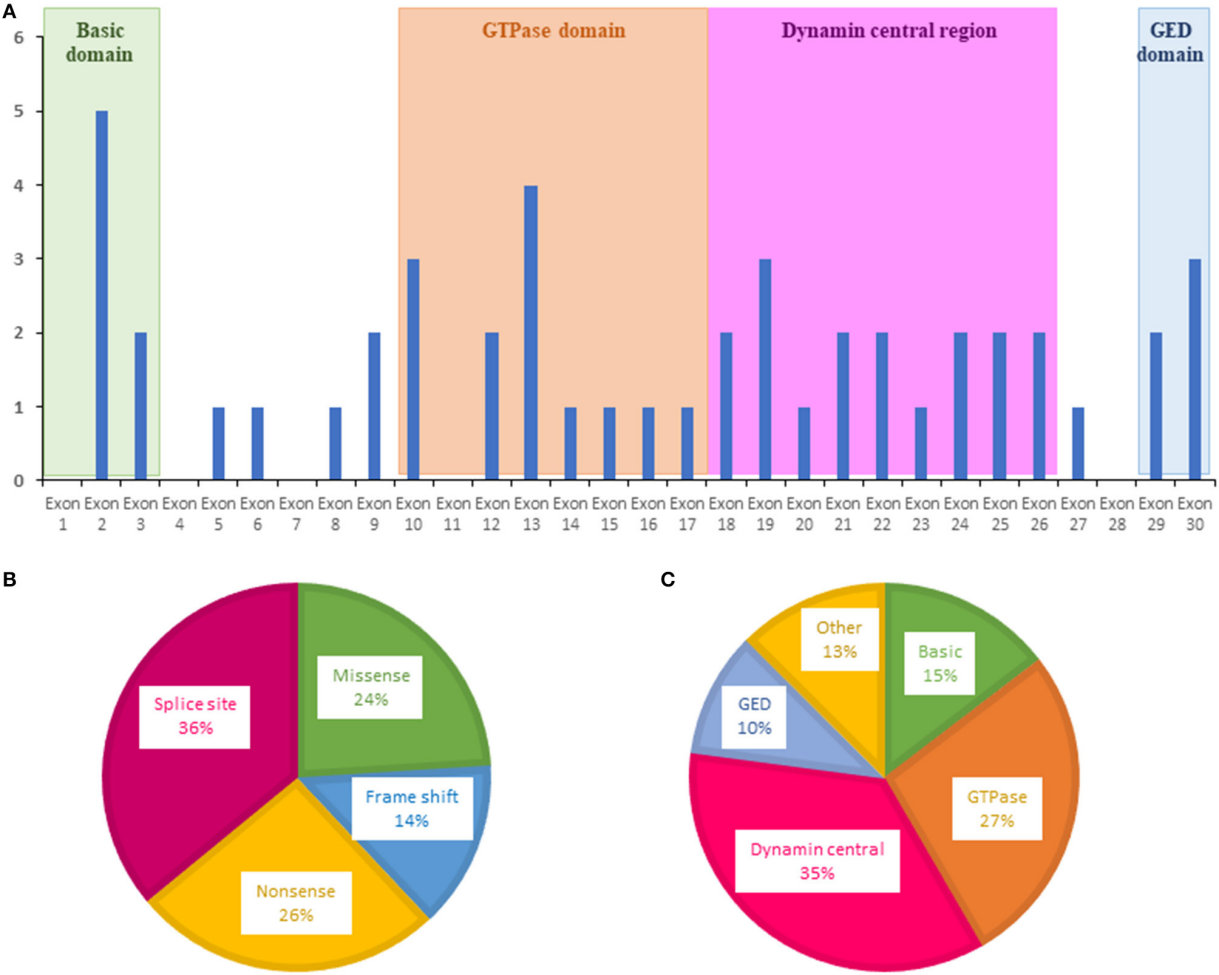
Distribution of variants identified in *OPA1* by the ION chip. **(A)** Exons in which were located the variants are shown as blue bars. **(B)** Types of variants. **(C)** Affected domain of the protein. GED, GTPase effector domain.

For *OPA1*, variants were more frequently evidenced in exons 2, 10, 13, and 19 (Figure 2A). The most frequent *OPA1* variants were splice variants (36%), followed by nonsense (26%), missense (24%), and frameshift variants (14%) (Figure 2B).

Regarding localization in the OPA1 protein, most of the variants were localized in the central GTPase (27%) and dynamin (35%) domains, highlighting the important role of these domains in OPA1 functions (Figure 2C).

### Recessive Genes

Twenty-three individuals (38.98%) out of 59 ROA cases were harboring biallelic pathogenic variants in *WFS1*. Regarding the other recessive genes, we found variants in *ACO2* in 13 families (22.03%), in *RTN4IP1* in 9 families (15.25%), and in *TMEM126A* or *SPG7* in 5 families each (8.47%). Finally, three families (5.08%) harbored *OPA1* biallelic variants, and one family, *SLC25A46* variants (Figure 1).

**Figure 3:**
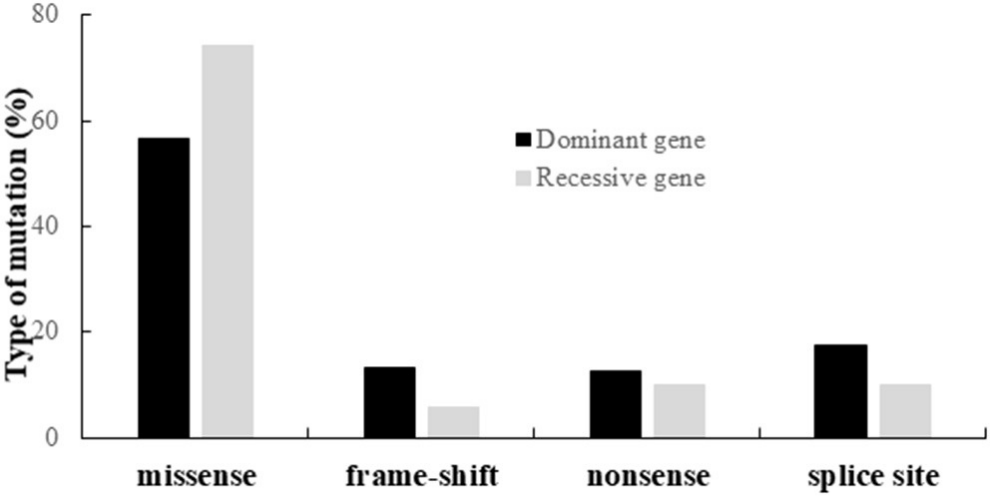
Distribution of the different types of variants identified in the genes responsible for dominant or recessive IONs.

Altogether, we identified 190 different variants, including 120 missenses, 22 nonsenses, 20 frameshifts, and 28 splice variants. The distribution of the different types of variants according to their dominant or recessive mode of inheritance is shown on Figure 3.

## Discussion

NGS is an effective approach that has dramatically improved the molecular diagnosis of genetic disorders, allowing a one-step identification of pathogenic variants in candidate genes. In this study, we have developed a panel for the molecular diagnosis of IONs, allowing the resequencing of 22 targeted genes, based on the Ion AmpliSeq method.

The development of this ION chip has improved the genetic dismemberment of IONs by allowing molecular diagnosis in a large number of cases and by contributing to the identification of new genes responsible for optic nerve degeneration.

Our results revealed that 22% of affected individuals (245 out of 1,102) harbored pathogenic variants in the targeted genes, 17% in the dominant form and 5% in the recessive form. The rather low ratio of success in inferring a molecular diagnosis from our study is most probably related to the fact that we provide a nationwide service to clinicians that might not always be familiar with the clinical diagnosis of ION.

Nevertheless, these results are in agreement with those of the literature indicating that DOAs are the most frequent forms of ION with an estimated frequency of 1/30,000 in the world (29). Conversely, the frequency of autosomal recessive optic neuropathies, which was initially described as very low, is now progressing, thanks to NGS, with an increasing number of genes involved (30).

In our cohort, we found that only 40.9% of the individuals with DOA presented a pathogenic heterozygous *OPA1* variant, which is lower than the involvement of this gene in 60–80% of individuals with DOA in the literature (31), which were collected among well-characterized family cases, whereas in our study most patients were isolated cases.

Most of these variants were located in the GTPase (27%) and the central dynamin (35%) domains, in accordance with data from the *OPA1* database, which includes 414 pathogenic variants (31).

Among the *OPA1* variants, 36% were associated with a splicing defect, 26% were nonsense, 14% were frameshift, and 24% were missense. This confirms that haploinsufficiency is more frequently involved than dominant negative variants in the pathomechanism of DOA (31, 32).

With the exception of *WFS1* and *SSBP1*, all proteins associated with DOA are closely related to mitochondrial dynamics, pointing to a key pathway that is essential for the maintenance and survival of RGC (17, 18, 33, 34). However, while excluding *OPA1*, the frequency of mutations in DOA that affect the proteins involved in mitochondrial dynamics is low. For example, *AFG3L2* is involved in only 4.3% of cases, *SPG7* in 3.23%, *MFN2* in 5.91%, and *DNM1L* or *MIEF1* in 1.08% of cases with a molecular diagnosis.

On the other hand, the results of ION chip sequencing revealed that *ACO2*, encoding the mitochondrial aconitase 2 involved in the Krebs cycle, is the second gene most frequently involved in DOA (16.67% of the cases). Variants in *ACO2* have until now been mostly involved in recessive IONs (25), but our results show that dominant cases are far more frequent than the recessive ones^1^. *ACO2* is therefore the third gene in order of frequency involved in autosomal IONs, after *OPA1* and *WFS1*^1^.

In this study, we also discovered dominant variants in five novel candidate genes (data not shown), suggesting that other mechanisms *a priori* complementary to mitochondrial dynamics are also involved in the pathophysiology of DOA.

As mentioned earlier, autosomal recessive IONs are less frequent. The first gene found to be involved in recessive ION was *TMEM126A*, which encodes a mitochondrial inner membrane protein (23). The most frequent mutation in *TMEM126A*, namely, p.R55X, was evidenced in five families (8.47%) of our series.

The other genes involved in recessive IONs are responsible for isolated or syndromic forms of optic atrophy. Recessive variants in *ACO2* were found in 13 families (22.03%), whereas nine families (15.25%) harbored *RTN4IP1* recessive variants. *RTN4IP1* encodes a mitochondrial protein with a quinone oxidoreductase activity, which is involved in complexes I and IV respiratory functions, and neuronal dendritic branching (22). For these three genes, mutated individuals had a clinical presentation that was, in most cases, restricted to isolated optic atrophy.

In contrast, the recessive variants identified in *SPG7* (8.47%), *OPA1* (5.08%), and *SLC25A46* (1.69%) were associated with syndromic optic atrophies.

In our series, variants in *WFS1* were found in 12.37 and 38.98% of dominant and recessive IONs, respectively, with most variants localized in exon 8 and leading to truncating and missense changes (18, 35–37). Mutations in *WFS1* are responsible for many different clinical presentations with dominant and recessive inheritance, including the Wolfram syndrome, autosomal recessive non-syndromic OA, isolated DOA (19), and DOA associated with hearing loss (18, 38).

The recurrent, non-founder mutation in *WFS1*, p.A684V, has been identified in six families from this series. This variant is a common cause of isolated autosomal DOA associated with deafness, as is the recurrent p.R500H mutation in *OPA1. WFS1* encodes the wolframin, a transmembrane endoplasmic reticulum protein that plays an essential role in calcium homeostasis and interorganelle cross-talk at mitochondria-associated membranes (33, 39), highlighting the important role of these structures in the pathophysiology of IONs.

## Conclusion

The NGS approach allowed us to identify the genetic cause of 22% of autosomal IONs. A total of 190 different variants were found in known genes and in five novel candidate genes. Further improvements in NGS technology, with a greater number of targeted genes, or the use of the whole-exome or whole-genome sequencing will undoubtedly increase the success rate of the molecular diagnosis of this group of diseases.

Finally, pathophysiological studies on the new genes involved in IONs will broaden our vision of the mechanisms responsible for the degeneration of CGRs and will eventually lead to new therapeutic strategies.

## Data Availability Statement

The datasets presented in this article are not readily available because they have been collected for official clinical molecular diagnosis performed at the University Hospital of Angers. Requests to access the datasets should be directed to GL and PA-B (guy.lenaers@univ-angers.fr and pabonneau@chu-angers.fr).

## Ethics Statement

The studies involving human participants were reviewed and approved by Institutional Review Board Committee of the University Hospital of Angers, Authorization number: AC-2012-1507. Written informed consent to participate in this study was provided by the participants' legal guardian/next of kin.

## Author Contributions

GL and PA-B: conception and design of the study. DB, EC, and AZ: recruitment of patients. MC, CB, DG, VD-D, PR, and PA-B: acquisition and data analysis. MC and GL: drafting a significant portion of the manuscript and figures. DB and VP: revised the manuscript for intellectual content. All authors contributed to the article and approved the submitted version.

## Conflict of Interest

The authors declare that the research was conducted in the absence of any commercial or financial relationships that could be construed as a potential conflict of interest.

## Footnotes

^1^Charif M, Gueguen N, Ferré M, Elkarhat Z, Khiati S, LeMao M, et al. Dominant ACO2 mutations are a frequent cause of isolated Optic Atrophy. *Brain Commun.* (in press).
